# CAND2/PMTR1 Is Required for Melatonin-Conferred Osmotic Stress Tolerance in Arabidopsis

**DOI:** 10.3390/ijms22084014

**Published:** 2021-04-13

**Authors:** Lin-Feng Wang, Ting-Ting Li, Yu Zhang, Jia-Xing Guo, Kai-Kai Lu, Wen-Cheng Liu

**Affiliations:** 1 State Key Laboratory of Crop Stress Adaptation and Improvement, State Key Laboratory of Cotton Biology, School of Life Sciences, Henan University, Kaifeng 475004, China; 15517110746@163.com (L.-F.W.); zhangyu2791@163.com (Y.Z.); august_0819@outlook.com (J.-X.G.); yhlkk_swkx@163.com (K.-K.L.); 2 Jiangsu Key Laboratory of Marine Pharmaceutical Compound Screening, Jiangsu Ocean University, Lianyungang 222005, China; littly2006@whu.edu.cn

**Keywords:** melatonin, osmotic stress, SNAT1, CAND2, ROS

## Abstract

Osmotic stress severely inhibits plant growth and development, causing huge loss of crop quality and quantity worldwide. Melatonin is an important signaling molecule that generally confers plant increased tolerance to various environmental stresses, however, whether and how melatonin participates in plant osmotic stress response remain elusive. Here, we report that melatonin enhances plant osmotic stress tolerance through increasing ROS-scavenging ability, and melatonin receptor CAND2 plays a key role in melatonin-mediated plant response to osmotic stress. Upon osmotic stress treatment, the expression of melatonin biosynthetic genes including *SNAT1*, *COMT1*, and *ASMT1* and the accumulation of melatonin are increased in the wild-type plants. The *snat1* mutant is defective in osmotic stress-induced melatonin accumulation and thus sensitive to osmotic stress, while exogenous melatonin enhances the tolerance of the wild-type plant and rescues the sensitivity of the *snat1* mutant to osmotic stress by upregulating the expression and activity of catalase and superoxide dismutase to repress H_2_O_2_ accumulation. Further study showed that the melatonin receptor mutant *cand2* exhibits reduced osmotic stress tolerance with increased ROS accumulation, but exogenous melatonin cannot revert its osmotic stress phenotype. Together, our study reveals that CADN2 functions necessarily in melatonin-conferred osmotic stress tolerance by activating ROS-scavenging ability in Arabidopsis.

## 1. Introduction

Melatonin (*N*-acetyl-5-methoxytryptamine) is a well-studied animal hormone synthesized and secreted by the pineal gland in mammals, but also serves as a vital signaling molecule in plants [1,2,3]. Melatonin plays important roles in the regulation of plant growth, development, and responses to various biotic and abiotic stresses including salt, drought, heavy metal, cold, heat, and pathogen infection [3,4,5]. Similar to that in animals, the melatonin biosynthesis pathway in plants starts from tryptophan, and is mainly produced by the activities of tryptophan decarboxylase (TDC), tryptophan 5-hydroxylase (T5H), serotonin *N*-acyltransferase (SNAT), and *N*-aceylserotonin *O*-methyltransferase (ASMT), caffeic acid *O*-methyltransferase (COMT) [6,7]. SNAT acts as the rate-limiting enzyme in the overall melatonin biosynthetic pathway in Arabidopsis [8], and its mutant is defective in UV-B or high light stress-induced melatonin accumulation, while its overexpressing transgenic plants have higher melatonin levels in the presence or even absence of stress treatment [8,9,10]. Recent study documented that the diurnal rhythmic oscillation pattern of melatonin accumulation in the wild-type plant was impaired in the *snat1* mutant [10].

Drought and high salinity are two major abiotic stresses adversely affecting plant growth and development, causing considerable losses in crop production and quality [11,12]. Previous reports widely documented that melatonin generally enhances plant drought and salt stress tolerance. For example, exogenous application of melatonin significantly enhances the drought tolerance of Arabidopsis, maize, Malus prunifolia, wheat, and rice plants [13,14,15,16,17,18,19], and also confers the salt tolerance of Arabidopsis, Malus zumi, alfalfa, tomato, sunflower, and rice plants [20,21,22,23,24,25,26]. Manipulating the in vivo melatonin content in various plants and then analyzing their drought or salt stress sensitivity also obtained a similar conclusion that melatonin can indeed improve plants drought and salt stress tolerance. For example, overexpressing melatonin biosynthetic gene *MzASMT1* or *MzSNAT5* of apple (*Malus zumi Mats*) in Arabidopsis greatly increases plant drought and salt stress tolerance with higher melatonin accumulation [17,19]. In addition, transgenic Arabidopsis plants overexpressing *MsSNAT* of alfalfa showed increased in vivo melatonin concentration and enhanced salinity tolerance [25]. Similarly, overexpressing the *COMT-like* gene *TaCOMT* of wheat (*Triticum aestivum L.*) in Arabidopsis results in elevated melatonin content and enhanced drought tolerance [18]. Additionally, overexpression transgenic tomato plants of *SlCOMT1* have higher melatonin content and increased salt stress tolerance [23]. Further, increasing melatonin concentration by suppressing the expression of *Melatonin 2-hydroxylase* (*M2H*) that catalyzes the conversion of melatonin into 2-hydroxymelatonin in rice led to decreased melatonin accumulation and salt stress tolerance [21].

Drought and high salinity induce similar effects on plants such as osmotic stress and oxidative damage but also elicit different effects on plants [12,27]. Plants have evolved as sophisticated and specific signaling pathway to sense and transduce osmotic stress signaling, which is distinct to the mechanism underlying plant response to ionic stress, oxidative damage, or drought soil-caused mechanical injury [28,29,30]. In addition to drought and salt, other environmental stresses such as cold and heavy metal also poses osmotic stress to plants [12]. However, how melatonin functions in plant osmotic stress response and tolerance remains to be further elucidated.

Unlike the detailed description of the melatonin receptor and signaling pathway established in mammals, the plant melatonin receptor was a mystery for a longtime until recent study identified the first phytomelatonin receptor CAND2/PMTR1 and analyzed its role in plant stomatal movement [31]. The in vitro radioligand-binding assay experiment indicates the binding of CAND2/PMTR1 to melatonin, and the impairment of melatonin-induced H_2_O_2_ accumulation in guar cells and stomatal closure further supports that CAND2 is a melatonin receptor in Arabidopsis [31]. Additionally, the *cand2/pmtr1* mutant loses diurnal stomatal closure with stomata remaining open during daytime and nighttime, revealing that CAND2/PMTR1 also plays a role in rhythmicity of phytomelatonin-regulated stomatal movement [10]. However, whether and how CAND2/PMTR1 participates in plant osmotic stress remain unknown.

In this study, we report that melatonin enhances plant osmotic stress tolerance and its receptor CAND2/PMTR1 plays a key role in melatonin-mediated plant response and tolerance to osmotic stress. When challenged with osmotic stress, the expression of melatonin biosynthetic gene *SNAT1* and receptor gene *CAND2* is increased in plants. Mutants of *cand2* and *snat1* with reduced melatonin accumulation are similarly sensitive to osmotic stress with lower antioxidant activities and increased ROS accumulation. Exogenous melatonin can markedly rescue the decreased osmotic stress tolerance of the *snat1* mutant for higher expression of genes encoding antioxidant enzymes, but these effects of melatonin on the *cand2* mutant were largely compromised. These results revealed that osmotic stress-induced *SNAT1* and *CAND2* function necessarily in the stress tolerance through the modulation of ROS scavenging ability in Arabidopsis.

## 2. Results

### 2.1. Melatonin Confers Plant Osmotic Stress Tolerance

**Figure 1:**
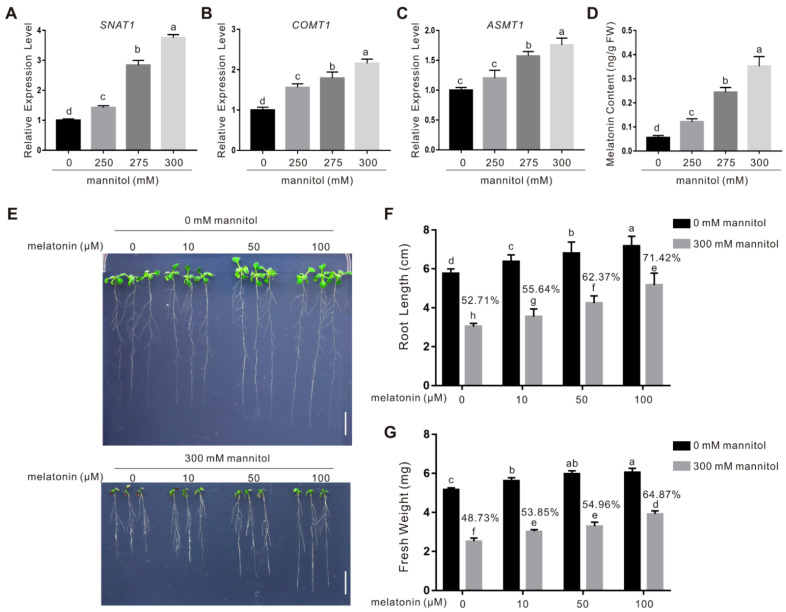
Melatonin enhances osmotic stress tolerance of the wild-type plants. (**A**–**C**) The expression of *SNAT1*, *COMT1*, and *ASMT1* in 5-day-old wild-type plant seedlings treated with 0, 250, 275, or 300 mM mannitol for 5 days was assayed by qPCR. The data are presented as means ± SD (*n* = 3). Bars with different letters indicate significant differences at *p* < 0.05, revealed using a one-way ANOVA with a Tukey’s multiple comparison test. *ACTIN2/8* was used as the reference gene. (**D**) Melatonin content in 5-day-old wild-type plant seedlings treated with 0, 250, 275, or 300 mM mannitol for 5 days. The data are presented as means ± SD from three biological replicates (*n* = 3). Bars with different letters indicate significant differences at *p* < 0.05, revealed using a one-way ANOVA with a Tukey’s multiple comparison test. Images of 5-day-old wild-type plant seedlings treated with or without 300 mM mannitol in the presence of 0, 10, 50, or 100 μM melatonin for 5 days. Bars = 1.0 cm. (**G**) Root length (**F**) and fresh weight (**G**) in (**E**). The percentages in the graphs indicate the relative root length (**F**) or fresh weight (**G**) by comparing those of mannitol-treated plants with those of untreated plants in the presence of a same concentration of melatonin. The data are presented as means ± SD from at least three independent experiments (*n* ≥ 30 for root length and *n* ≥ 10 for fresh weight). Bars with different letters indicate significant differences at *p* < 0.05, revealed using a one-way ANOVA with a Tukey’s multiple comparison test.

Universal roles of melatonin in plan diverse biotic and abiotic stresses have been widely documented, while whether and how melatonin participates in plant response to osmotic stress have not been fully elucidated. To investigate this, the wild-type Arabidopsis plant seedlings were treated with osmotic stress caused by high concentrations of mannitol, and the expression of *SNAT1*, *COMT1*, and *ASMT1*, three key genes involved in melatonin biosynthesis, was determined using qPCR. Our results showed that upon osmotic stress exposure, the expression of *SNAT1*, *COMT1*, and *ASMT1* was significantly induced in the wild-type plants (Figure 1A–C), suggesting the involvement of melatonin biosynthesis in plant response to osmotic stress. Thus, we directly assayed the concentrations of melatonin in plants treated with or without osmotic stress. Indeed, melatonin accumulation was extensively promoted by osmotic stress in a mannitol dosage-dependent manner (Figure 1D), which is consistent with the effect of osmotic stress on the expression of melatonin biosynthesis genes.

To study the role of melatonin in plant osmotic stress tolerance, we examined the effect of melatonin on plant osmotic stress tolerance by analyzing root elongation and fresh weight. Five-day-old wild-type seedlings were transferred onto 1/2 MS medium containing with or without 300 mM mannitol and different concentrations of melatonin, and grew vertically for another 5 days. Our results showed that mannitol-produced osmotic stress strongly suppressed plant primary root elongation and fresh weight while melatonin markedly promoted plant tolerance as evidenced by longer root and higher fresh weight of the treated plants (Figure 1E–G). Although exogenous melatonin also promoted root elongation and fresh weight accumulation under non-stress conditions which is consistent with a previous report [32], it elicited a more profound effect on osmotic stressed plants than the non-stressed control (Figure 1E–G), indicating that exogenous melatonin can confer plants increased osmotic stress tolerance.

**Figure 2:**
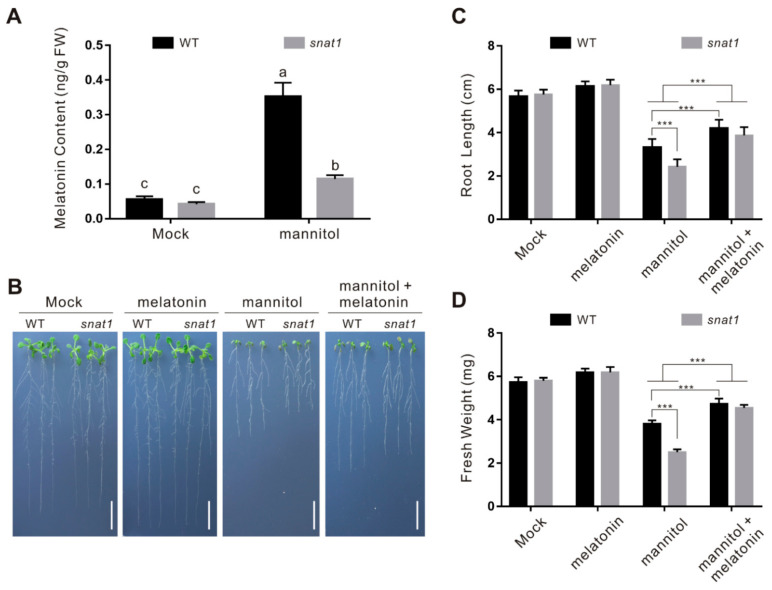
The snat1 mutant is sensitive to osmotic stress. (**A**) Melatonin content in 5-day-old wild-type and *snat1* mutant seedlings treated with or without 300 mM mannitol for 5 days. The data are presented as means ± SD from three biological replicates (*n* = 3). Bars with different letters indicate significant differences at *p* < 0.05, revealed using a one-way ANOVA with a Tukey’s multiple comparison test. Images of 5-day-old wild-type and snat1 mutant seedlings treated with or without 300 mM mannitol in the presence or absence of 100 μM melatonin for 5 days. Bars = 1.0 cm. (**C**,**D**) Root length (**C**) and fresh weight (**D**) in (**B**). The data are presented as means ± SD from at least three independent experiments (*n* ≥ 30 for root length and *n* ≥ 10 for fresh weight). Asterisks indicate significant differences using a Student’s *t*-test, *** *p* < 0.001.

To dissect the role of endogenous melatonin in plant osmotic stress tolerance, *snat1* mutant was employed and subjected to osmotic stress treatment as previous reports showed that the mutant has impaired melatonin biosynthesis and thus decreased melatonin accumulation [8]. We first verified whether osmotic stress-induced melatonin accumulation was defective in the *snat1* mutant. Our results showed that osmotic stress-induced melatonin accumulation in the wild-type plant was extremely repressed in the *snat1* mutant (Figure 2A). Next, the wild-type and *snat1* mutant seedlings were subjected to treatments of 300 mM mannitol and different concentrations of melatonin. Our results showed that both the wild-type and *snat1* mutant seedlings had similar root length and fresh weight under control conditions, while osmotic stressed *snat1* mutant had shorter roots and lower fresh weight than the treated wild-type plants (Figure 2B–D), indicating that the *snat1* mutant was more sensitive to osmotic stress than the wild-type plant. These data suggested that decreased tolerance of the *snat1* mutant to osmotic stress might be due to melatonin deficiency. If this was the case, exogenously applying melatonin to *snat1* mutant should rescue the mutant phenotype to the osmotic stress. Therefore, we assessed the sensitivity of the *snat1* mutant to osmotic stress in the presence of melatonin, and found that, increased osmotic stress sensitivity of the *snat1* mutant was nearly completely reverted by the exogenous melatonin (Figure 2B–D), further supporting the role of melatonin in enhancing plant osmotic stress tolerance in vitro and in vivo.

### 2.2. Melatonin Functions in Plant Osmotic Stress Tolerance through Regulating ROS Homeostasis

**Figure 3:**
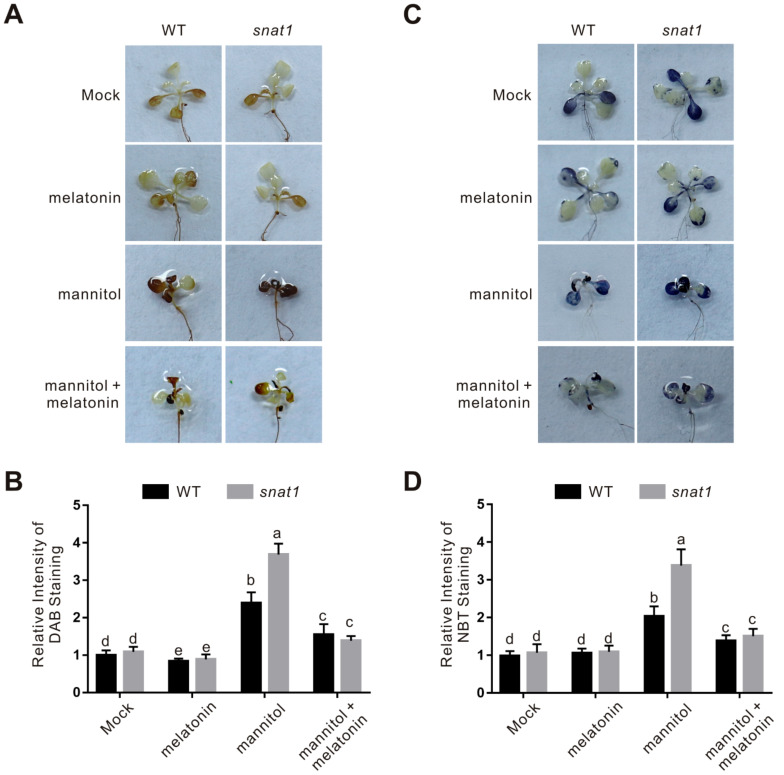
Reactive oxygen species (ROS) accumulation in the wild-type and *snat1* mutant seedlings. (**A**) The DAB-staining images of leaves from 5-day-old wild-type and *snat1* mutant seedlings treated with 300 mM mannitol in the presence or absence of 100 μM melatonin for 5 days. (**B**) The relative DAB-staining intensity in (**A**). The DAB staining intensity of wild-type leaves without treatment was set to 1. The data are presented as means ± SD from at least three biological replicates (*n* = 10). Bars with different letters indicate significant differences at *p* < 0.05, revealed using a one-way ANOVA with a Tukey’s multiple comparison test. (**C**) The NBT-staining images of leaves from 5-day-old wild-type and *snat1* mutant seedlings treated with 300 mM mannitol in the presence or absence of 100 μM melatonin for 5 days. (**D**) The relative NBT-staining intensity in (**C**). The NBT staining intensity of wild-type leaves without treatment was set to 1. The data are presented as means ± SD from at least three biological replicates (*n* ≥ 10). Bars with different letters indicate significant differences at *p* < 0.05, revealed using a one-way ANOVA with a Tukey’s multiple comparison test.

Melatonin enhances plant osmotic tress tolerance, while the underlying mechanism remain elusive. Previous reports documented that melatonin functions in plant various abiotic stresses partially by enhancing plant ROS scavenging ability. Thus, we assayed the ROS accumulation in the wild-type and *snat1* mutant seedlings treated with osmotic stress in the presence or absence of melatonin. The 3,3-diaminobenzidine (DAB) staining experiment was employed to indicate the H_2_O_2_ accumulation in plants, and our results showed that both the stress-treated wild-type and *snat1* mutant plants had increased H_2_O_2_ accumulation compared with their untreated control, while the H_2_O_2_ accumulation in the stressed mutant was significantly higher than that in the wild-type plants (Figure 3A,B). We also examined the superoxide anion accumulation by performing nitrioblue tetrazolium (NBT) staining assay. Our results showed that, similarly to H_2_O_2_ accumulation, the *snat1* mutant also had higher superoxide anion accumulation than the wild-type plant when treated with osmotic stress (Figure 3C,D). These results reveal a role of melatonin in repressing osmotic stress-induced ROS accumulation in plants. In addition, consistent with the effect of exogenous melatonin on plant osmotic stress tolerance, we also found that increased H_2_O_2_ and superoxide anion accumulation in the stressed wild-type plant was significantly repressed by exogenously applied melatonin, and that higher ROS accumulation in the osmotic stressed *snat1* mutant was also decreased to about the level of the wild-type plant in the presence of melatonin (Figure 3), further supporting the key role of melatonin in scavenging osmotic stress-induced ROS accumulation in plants.

**Figure 4:**
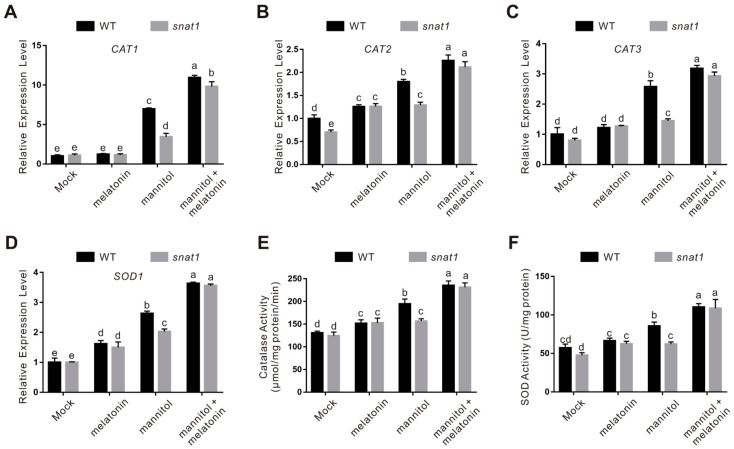
The expression and activity of catalase and superoxide dismutase (SOD) in the wild-type and *snat1* mutant seedlings. (**A**–**D**) The expression of *CAT1* (**A**), *CAT2* (**B**), *CAT3* (**C**), and *SOD1* (**D**) in 5-day-old wild-type and *snat1* mutant seedlings treated with 300 mM mannitol in the presence or absence of 100 μM melatonin for 5 days was assayed by qPCR. The data are presented as means ± SD (*n* = 3). Bars with different letters indicate significant differences at *p* < 0.05, revealed using a one-way ANOVA with a Tukey’s multiple comparison test. *ACTIN2/8* was used as the reference gene. (**E**,**F**) The catalase (**E**) and SOD (**F**) activities in 5-day-old wild-type and *snat1* mutant seedlings treated with 300 mM mannitol in the presence or absence of 100 μM melatonin for 5 days. The data are presented as means ± SD from at least three biological replicates (*n* ≥ 3). Bars with different letters indicate significant differences at *p* < 0.05, revealed using a one-way ANOVA with a Tukey’s multiple comparison test.

To further investigate the role of melatonin in regulating osmotic stress-induced ROS accumulation, expression of key genes involved in the ROS scavenging system such as catalase (CAT) and superoxide dismutase (SOD) was assessed. Our qPCR results showed that the expression of *CAT1*, *CAT2*, *CAT3*, and *SOD1* in the *snat1* mutant was significantly lower than that in the wild-type plants when subjected to osmotic stress, while melatonin treatment further upregulated their expression both in the wild-type and *snat1* mutant plants (Figure 4A–D). In line with this, catalase and SOD activities in the stressed *snat1* mutant was lower than that in the treated wild-type plant, and exogenously applied melatonin promoted these activities both in the wild-type and mutant (Figure 4E,F). Together, these results clearly indicate that SNAT1-mediated melatonin biosynthesis contributes to plant osmotic stress tolerance at least partially through promoting ROS scavenging activities.

### 2.3. CAND2/PMTR1 Is an Osmotic Stress-Responsive Gene

**Figure 5:**
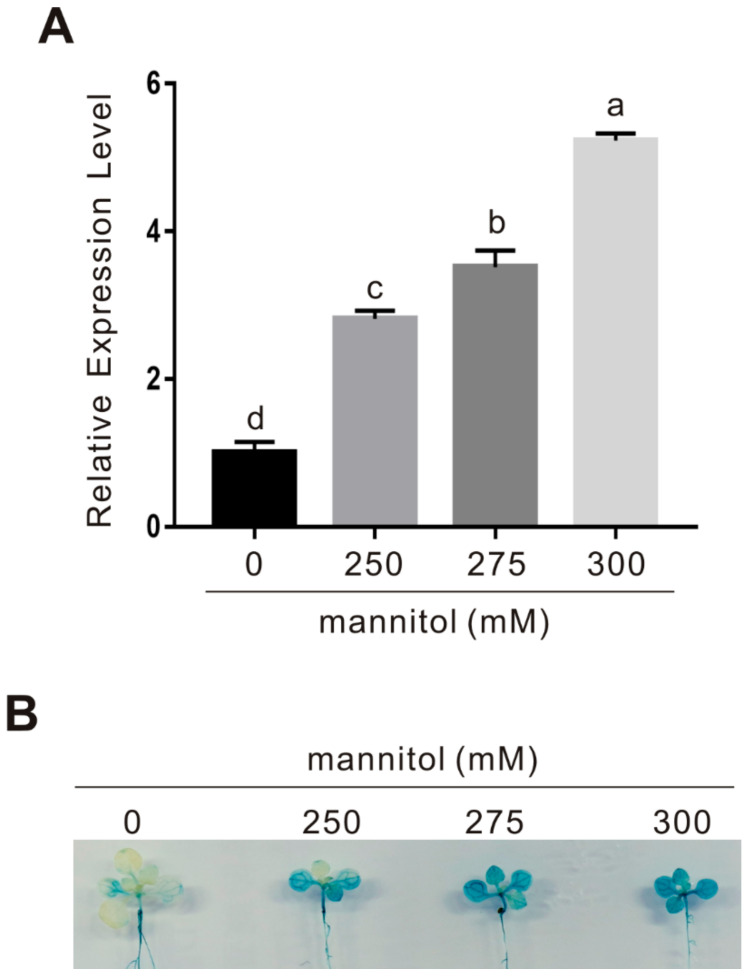
Osmotic stress upregulates *CAND2* expression in plants. (**A**) The expression of *CAND2* in 10-day-old wild-type plant seedlings treated with or without 250, 275, or 300 mM mannitol for 5 days was assayed by qPCR. The data are presented as means ± SD (*n* = 3). Bars with different letters indicate significant differences at *p* < 0.05, revealed using a one-way ANOVA with a Tukey’s multiple comparison test. ACTIN2/8 was used as the reference gene. (**B**) The GUS-staining images of leaves from 10-day-old *CAND2::GUS* transgenic plant seedlings treated with 0, 250, 275, or 300 mM mannitol for 12 h.

Our above results reveal that melatonin increases plant osmotic stress tolerance by upregulating the expression of some antioxidant enzymes in vitro and in vivo, while how melatonin functions in this process remains to be further elucidated. Recent progress about melatonin receptor CAND2/PMTR1 in Arabidopsis prompts us to further investigate whether CAND2/PMTR1 participates in melatonin-conferred plant osmotic stress tolerance. We first examined the expression of *CAND2* in osmotic stress-treated plants, and our qPCR result showed that the transcription level of *CAND2* in the wild-type plant was significantly upregulated by mannitol in a dosage-dependent manner (Figure 5A). This note was confirmed using *CAND2::GUS* transgenic plants treated with or without osmotic stress, and we found that the GUS staining in stressed plants was obviously darker than that in the non-stressed control plant (Figure 5B), supporting that osmotic stress induces *CAND2* expression. These results imply that the melatonin receptor CAND2 is an osmotic stress-responsive gene.

### 2.4. CAND2/PMTR1 Participates in Melatonin-Conferred Osmotic Stress Tolerance in Plants

**Figure 6:**
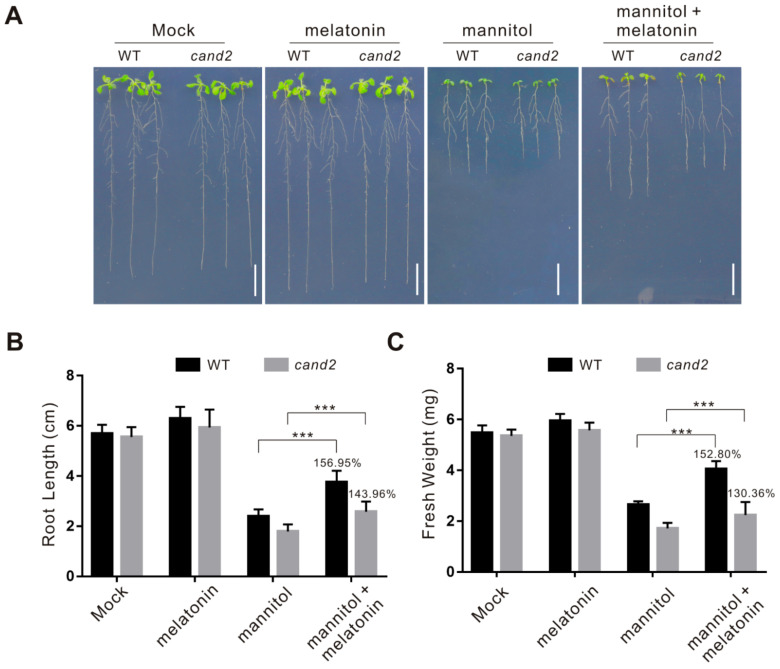
The *cand2* mutant is sensitive to osmotic stress. (**A**) Images of 5-day-old wild-type and *cand2* mutant seedlings treated with or without 300 mM mannitol in the presence or absence of 100 μM melatonin for 5 days. Bars = 1.0 cm. (**B**,**C**) Root length (**B**) and fresh weight (**C**) in (**A**). The percentages in the graphs indicate the relative root length (**B**) or fresh weight (**C**) by comparing those of mannitol plus melatonin-treated plants with those of mannitol-treated plants in the presence of mannitol. The data are presented as means ± SD from at least three independent experiments (*n* ≥ 30 for root length and *n* ≥ 10 for fresh weight). Asterisks indicate significant differences using a Student’s *t*-test (*** *p* < 0.001).

To study the role of CAND2 in plant osmotic stress tolerance, we then tested the sensitivity of the *cand2* mutant to the stress. The *cand2* mutant had similar primary root length and fresh weight with the wild-type plant under normal conditions, however, when subjected to osmotic stress, the *cand2* mutant displayed increased sensitivity compared with the wild-type plant as evidenced by shorter root and lower fresh weight (Figure 6), indicating that melatonin receptor CAND2 plays a role in plant osmotic stress tolerance.

**Figure 7:**
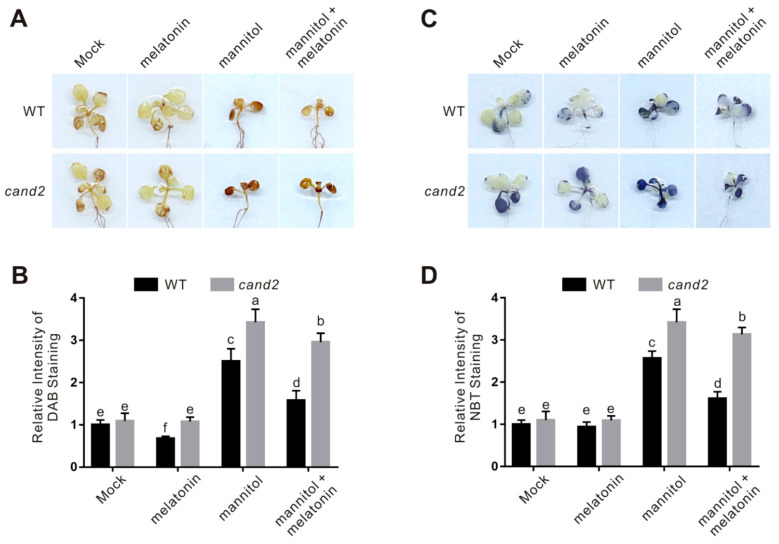
ROS accumulation in the wild-type and *cand2* mutant seedlings. (**A**) The DAB-staining images of leaves from 5-day-old wild-type and *cand2* mutant seedlings treated with 300 mM mannitol in the presence or absence of 100 μM melatonin for 5 days. (**B**) The relative DAB-staining intensity in (**A**). The DAB staining intensity of wild-type leaves without treatment was set to 1. The data are presented as means ± SD from at least three biological replicates (*n* ≥ 10). Bars with different letters indicate significant differences at *p* < 0.05, revealed using a one-way ANOVA with a Tukey’s multiple comparison test. (**C**) The NBT-staining images of leaves from 5-day-old wild-type and *cand2* mutant seedlings treated with 300 mM mannitol in the presence or absence of 100 μM melatonin for 5 days. (**D**) The relative NBT-staining intensity in (**C**). The NBT staining intensity of the wild-type leaves without treatment was set to 1. The data are presented as means ± SD from at least three biological replicates (*n* ≥ 10). Bars with different letters indicate significant differences at *p* < 0.05, revealed using a one-way ANOVA with a Tukey’s multiple comparison test.

**Figure 8:**
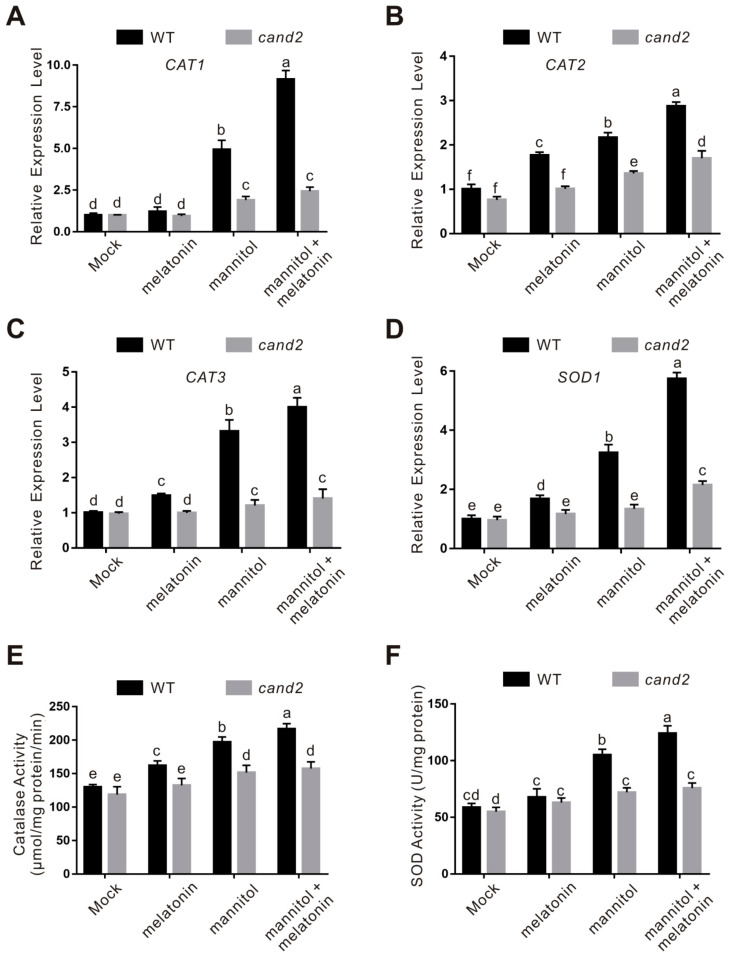
The expression and activity of catalase and SOD in the wild-type and *cand2* mutant seedlings. (**A**–**D**) The expression of *CAT1* (**A**), *CAT2* (**B**), *CAT3* (**C**), and *SOD1* (**D**) in 5-day-old wild-type and *cand2* mutant seedlings treated with 300 mM mannitol in the presence or absence of 100 μM melatonin for 5 days was assayed by qPCR. The data are presented as means ± SD (*n* = 3). Bars with different letters indicate significant differences at *p* < 0.05, revealed using a one-way ANOVA with a Tukey’s multiple comparison test. *ACTIN2/8* was used as the reference gene. (**E**,**F**) The catalase (**E**) and SOD (**F**) activities in 5-day-old wild-type and *cand2* mutant seedlings treated with 300 mM mannitol in the presence or absence of 100 μM melatonin for 5 days. The data are presented as means ± SD from at least three biological replicates (*n* ≥ 3). Bars with different letters indicate significant differences at *p* < 0.05, revealed using a one-way ANOVA with a Tukey’s multiple comparison test.

Considering that melatonin increases plant osmotic stress tolerance by activating ROS scavenging activities, we further examined the accumulation of H_2_O_2_ and superoxide anion in the *cand2* mutant by DAB and NBT staining, respectively. Similar to the *snat1* mutant, accumulation of H_2_O_2_ and superoxide in osmotic stressed *cand2* mutant was significantly higher than that in the treated wild-type plants (Figure 7). In addition, osmotic stress-induced expression of the key genes encoding ROS-scavenging enzymes including *CAT1*, *CAT2*, *CAT3*, and *SOD1* in the wild-type plant was largely compromised in the *cand2* mutant (Figure 8A–D). Consistently, when treated with osmotic stress, both catalase and SOD activities in the *cand2* mutant was repressed compared with the wild-type plant (Figure 8E,F). These results indicated that CAND2 functions in plant osmotic stress tolerance by enhancing ROS scavenging ability in a similar way to melatonin.

To dissect whether CAND2 is involved in melatonin-conferred plant osmotic stress tolerance, melatonin was exogenously applied to the wild-type and cand2 mutant plant seedlings. Our results showed that melatonin significantly increased the tolerance of the wild-type plant to osmotic stress in terms of root length and fresh weight, but these effects were largely dampened in the cand2 mutant (Figure 6), suggesting the involvement of CAND2 in melatonin action in plant osmotic stress tolerance. This note was reinforced by assaying the role of CAND2 in melatonin-induced ROS-scavenging ability. Our results showed that melatonin strongly induced the transcription and activities of catalase and SOD in the wild-type plant but not in the cand2 mutant in the presence of osmotic stress (Figure 7 and Figure 8). Together, these data indicate that CAND2 is required for melatonin-conferred osmotic stress tolerance in Arabidopsis.

## 3. Discussion

Melatonin functions in plant responses to various environmental stresses including drought, salt, heat, clod, heavy metal, UV radiation, and pathogen infection [2,3], however, its role in osmotic stress has not been studied exactly. In this study, we showed that melatonin promotes osmotic stress tolerance by conferring plants higher ROS-scavenging ability, which is consistent with the mechanism of melatonin in other biotic stresses such as high salinity, heavy metal, and UV radiation [3,4,33,34]. Previous reports documented that melatonin plays its role in degrading ROS as a natural antioxidant to regulate ROS homeostasis while it is also observed in previous reports and in our study here that melatonin could significantly upregulate the expression of ROS-metabolizing-related genes such as CATs, SODs, and APXs, suggesting a regulatory role of melatonin signaling in plant response to stresses. Our results showed that osmotic stress-induced expression of CAT1, CAT2, CAT3, and SOD1 in the wild-type plants was largely inhibited in the snat1 and cand2 mutant, while exogenous melatonin could rescue the expression of these genes in the melatonin-synthesis mutant snat1 but not in the melatonin receptor mutant cand2, revealing a necessary role of CAND2 in melatonin-mediated regulation of gene expression. Consistent with this, osmotic stress-induced ROS over-accumulation including H2O2 and superoxide in the snat1 mutant could be well reverted to that of the wild-type plants, but in cand2 mutant, melatonin could not exert its effect, further supporting the role of CAND2 in melatonin-mediated signaling transduction in Arabidopsis. Additionally, we also noticed that exogenous melatonin mildly increased osmotic stress tolerance of the cand2 mutant in terms of root length and fresh weight with decreased ROS accumulation, which is possibly due to the ROS-scavenging ability of melatonin as a natural potent ROS scavenger [35].

It has not been reported the downstream players of CAND2 in melatonin-mediated plant stress response so far. As a plasma-membrane protein, CAND2 recognizes and binds melatonin at the plasma-membrane [31], however, how CAND2 transfers melatonin signaling to nucleus and modulates gene expression is worthy to be further explored. Screening and identification of CAND2-interacting proteins may shed some light on the mechanism underlying-melatonin and its receptor-mediated signaling transduction in plant response to osmotic stress.

Considering the role of melatonin in various abiotic stresses, we speculated that CAND2 may functions in other environmental stresses in a similar way to that in the osmotic stress. We also noticed that osmotic stress upregulates the expression of *CAND2* and melatonin biosynthetic genes such as *SNAT1*, *COMT1*, and *ASMT1*. Increased accumulation of melatonin and melatonin receptor CAND2 synergistically can contribute to melatonin signaling transduction and thus further promoting plant osmotic stress tolerance. How plants sense osmotic stress and thus regulating the expression of *CAND2* and melatonin biosynthetic genes remains unknown, and is also worthy to be experimentally investigated.

## 4. Materials and Methods

### 4.1. Plant Material and Growth Conditions

Arabidopsis (*Arabidopsis thaliana*) ecotype Columbia was used in this study. The *snat1* (SALK_032239) and *cand2* (SALK_071302C) mutant and *CAND2::GUS* seeds were previously reported [31,36]. Seeds were surface sterilized for 5 min in 5% commercial bleach, washed three times with sterile water, and plated on half strength Murashige and Skoog (MS) medium (pH 5.8) (Sigma-Aldrich, St. Louis, MO, USA) containing 1% sucrose and 1% agar. Plants were stratified at 4 °C for 3 d in the dark, and then transferred to chambers. The seedlings grown vertically at 22 °C and 100 µmol m^−2^ s^−1^ illumination under 16 h light/8 h dark conditions for 5 days were transferred to 1/2 MS medium without or with different concentrations of mannitol (Sigma-Aldrich) for another 5 days, and then the root length and fresh weight were measured and analyzed.

### 4.2. 3,3-diaminobenzidine (DAB) Staining and Nitrioblue Tetrazolium (NBT) Staining

The 5-day-old seedlings were transferred to half strength 1/2 MS medium without or with 300 mM mannitol for 5 days, and then used for DAB or NBT staining to assay H_2_O_2_ or superoxide anion accumulation as we described previously [37,38,39]. For DAB staining, the seedlings were incubated in freshly prepared DAB staining solution (1 mg/mL DAB and 0.1% Tween 20 in 10 mM Na_2_HPO_4_) for 8 h, and then rinsed with 70% ethanol for several times to remove the chlorophyll. The images of the leaves were captured using a digital camera. For superoxide anion staining, the seedlings were vacuum infiltrated with 0.1 mg/mL NBT in 25 mM HEPES buffer (pH 7.6) for 2 h in darkness. Chlorophyll was removed by using 70% ethanol, and then the images of the leaves were captured using a digital camera. Three independent biological replicates were performed, and the relative intensity of DAB or NBT staining was quantitatively analyzed according to our previously reported method [37].

### 4.3. Detection of Catalase (CAT) and Superoxide Dismutase (SOD) Activity

Osmotic stress-treated or untreated seedlings were ground to fine powder under liquid nitrogen, and suspended in cold protein extraction buffer (50 mM potassium phosphate buffer, pH 7.8, 0.2 mM EDTA-Na_2_, 0.1 mM ascorbic acid, and 1% PVPP). After centrifugation at 12,000 g for 10 min at 4 °C, the supernatant was transferred to a new tube for further use. The protein concentration was assayed by the Bradford method, and CAT or SOD activity was determined according to the published methods [10,40,41]. CAT activity was assayed by monitoring the consumption of H_2_O_2_ at 240 nm [41]. One unit of SOD activity was defined as the quantity of crude protein extracts required to produce 50% inhibition of NBT reduction under the experimental conditions [40].

### 4.4. Quantitative Real-Time PCR

Treated or untreated seedlings were collected for total RNA isolation, first-strand cDNA synthesis, and qRT-PCR as we described previously [42]. The constitutively expressed ACTIN2/8 gene was used as an internal control. All experiments were repeated at least three times. The primer sequences are listed in Table S1.

### 4.5. Melatonin Extraction and Assay

Melatonin of the osmotic stress-treated and untreated seedlings was extracted and measured using an immunoassay kit (#ml036336, Shanghai Enzyme-linked Biotechnology Co., Ltd.; Shanghai, China) according to the manufacturer’s instructions. The OD values of the samples were recorded by reading spectrophotometric absorbance at wavelength of 450 nm (CLARIOstar PLUS, BMG LABTECH, Ortenberg, Germany), and the melatonin contents of the samples were calculated based on a standard curve of melatonin.

### 4.6. β-glucuronidase (GUS) Staining

The GUS histochemical staining experiment was performed as we previously described [37]. Ten-day-old CAND2::GUS transgenic seedlings were treated with 250, 275, or 300 mM mannitol for 12 h, and incubated at 37 °C in GUS staining solution (100 mM sodium phosphate buffer, pH 7.5, 10 mM EDTA, 0.5 mM potassium ferricyanide, 0.5 mM potassium ferrocyanide, 1 mM 5-bromochloro-3-indolyl-b-d-glucuronide, and 0.1% Triton X-100). Then, the leaves were rinsed with 70% ethanol for several times to remove the chlorophyll, and images were captured using a digital camera.

### 4.7. Statistical Analysis

Data are means (±SD) of three biological replicates, and the asterisks indicate statistically significant differences (* *p* < 0.05, ** *p* < 0.01, and *** *p* < 0.001, Student’s *t*-test). Bars with different letters indicate significant differences at *p* < 0.05 by two-way ANOVA with Tukey’s multiple comparison test.

## Data Availability

Data is contained within the article or Supplementary Materials.

## Supplementary Materials

The following are available online at https://www.mdpi.com/article/10.3390/ijms22084014/s1.

## References

[1] Arnao M.B., Hernandez-Ruiz J. (2020). Is Phytomelatonin a New Plant Hormone?. Agronomy.

[2] Arnao M.B., Hernandez-Ruiz J. (2019). Melatonin: A New Plant Hormone and/or a Plant Master Regulator?. Trends Plant Sci..

[3] Sun C., Liu L., Wang L., Li B., Jin C., Lin X. (2021). Melatonin: A master regulator of plant development and stress responses. J. Integr. Plant Biol..

[4] Bajwa V.S., Shukla M.R., Sherif S.M., Murch S.J., Saxena P.K. (2014). Role of melatonin in alleviating cold stress in *Arabidopsis thaliana*. J. Pineal. Res..

[5] Hernandez-Ruiz J., Cano A., Arnao M.B. (2005). Melatonin acts as a growth-stimulating compound in some monocot species. J. Pineal. Res..

[6] Wei Y., Bai Y., Cheng X., Zhu B., Reiter R.J., Shi H. (2020). The dual roles of melatonin biosynthesis enzymes in the coordination of melatonin biosynthesis and autophagy in cassava. J. Pineal. Res..

[7] Wei Y., Bai Y., Cheng X., Reiter R.J., Yin X., Shi H. (2021). Lighting the way: Advances in transcriptional regulation and integrative crosstalk of melatonin biosynthetic enzymes in cassava. J. Exp. Bot..

[8] Yao J.W., Ma Z., Ma Y.Q., Zhu Y., Lei M.Q., Hao C.Y., Chen L.Y., Xu Z.Q., Huang X. (2021). Role of melatonin in UV-B signaling pathway and UV-B stress resistance in *Arabidopsis thaliana*. Plant Cell Environ..

[9] Lee H.Y., Back K. (2018). Melatonin induction and its role in high light stress tolerance in *Arabidopsis thaliana*. J. Pineal. Res..

[10] Li D., Wei J., Peng Z., Ma W., Yang Q., Song Z., Sun W., Yang W., Yuan L., Xu X. (2020). Daily rhythms of phytomelatonin signaling modulate diurnal stomatal closure via regulating reactive oxygen species dynamics in Arabidopsis. J. Pineal. Res..

[11] Wang P., Sun X., Li C., Wei Z.W., Liang D., Ma F.W. (2013). Long-term exogenous application of melatonin delays drought-induced leaf senescence in apple. J. Pineal. Res..

[12] Zhu J.K. (2016). Abiotic Stress Signaling and Responses in Plants. Cell.

[13] Huang B., Chen Y.E., Zhao Y.Q., Ding C.B., Liao J.Q., Hu C., Zhou L.J., Zhang Z.W., Yuan S., Yuan M. (2019). Exogenous Melatonin Alleviates Oxidative Damages and Protects Photosystem II in Maize Seedlings Under Drought Stress. Front. Plant Sci..

[14] Li C., Tan D.X., Liang D., Chang C., Jia D., Ma F. (2015). Melatonin mediates the regulation of ABA metabolism, free-radical scavenging, and stomatal behaviour in two *Malus* species under drought stress. J. Exp. Bot..

[15] Liang D., Ni Z., Xia H., Xie Y., Lv X., Wang J., Lin L., Deng Q., Luo X. (2019). Exogenous melatonin promotes biomass accumulation and photosynthesis of kiwifruit seedlings under drought stress. Sci. Hortic..

[16] Niu X., Deqing C., Liang D. (2019). Effects of exogenous melatonin and abscisic acid on osmotic adjustment substances of ‘Summer Black’ grape under drought stress. Iop Conf..

[17] Wang L., Feng C., Zheng X., Guo Y., Zhou F., Shan D., Liu X., Kong J. (2017). Plant mitochondria synthesize melatonin and enhance the tolerance of plants to drought stress. J. Pineal. Res..

[18] Yang W.J., Du Y.T., Zhou Y.B., Chen J., Xu Z.S., Ma Y.Z., Chen M., Min D.H. (2019). Overexpression of TaCOMT Improves Melatonin Production and Enhances Drought Tolerance in Transgenic Arabidopsis. Int. J. Mol. Sci..

[19] Zuo B., Zheng X., He P., Wang L., Lei Q., Feng C., Zhou J., Li Q., Han Z., Kong J. (2014). Overexpression of MzASMT improves melatonin production and enhances drought tolerance in transgenic *Arabidopsis thaliana* plants. J. Pineal. Res..

[20] Chen L., Liu L., Lu B., Ma T., Li C. (2020). Exogenous melatonin promotes seed germination and osmotic regulation under salt stress in cotton (*Gossypium hirsutum* L.). PLoS ONE.

[21] Choi G.H., Back K. (2019). Suppression of Melatonin 2-Hydroxylase Increases Melatonin Production Leading to the Enhanced Abiotic Stress Tolerance against Cadmium, Senescence, Salt, and Tunicamycin in Rice Plants. Biomolecules.

[22] Liang C., Zheng G., Li W., Wang Y., Hu B., Wang H., Wu H., Qian Y., Zhu X.G., Tan D.X. (2015). Melatonin delays leaf senescence and enhances salt stress tolerance in rice. J. Pineal. Res..

[23] Liu D.D., Sun X.S., Liu L., Shi H.D., Chen S.Y., Zhao D.K. (2019). Overexpression of the Melatonin Synthesis-Related Gene SlCOMT1 Improves the Resistance of Tomato to Salt Stress. Molecules.

[24] Mukherjee S., David A., Yadav S., Baluska F., Bhatla S.C. (2014). Salt stress-induced seedling growth inhibition coincides with differential distribution of serotonin and melatonin in sunflower seedling roots and cotyledons. Physiol. Plant.

[25] Zhao G., Yu X.L., Lou W., Wei S.Q., Wang R., Wan Q., Shen W.B. (2019). Transgenic Arabidopsis overexpressing MsSNAT enhances salt tolerance via the increase in autophagy, and the reestablishment of redox and ion homeostasis. Environ. Exp. Bot..

[26] Zheng X., Tan D.X., Allan A.C., Zuo B., Zhao Y., Reiter R.J., Wang L., Wang Z., Guo Y., Zhou J. (2017). Chloroplastic biosynthesis of melatonin and its involvement in protection of plants from salt stress. Sci. Rep..

[27] Yang Y., Guo Y. (2018). Elucidating the molecular mechanisms mediating plant salt-stress responses. New Phytol..

[28] Chen K., Gao J., Sun S., Zhang Z., Yu B., Li J., Xie C., Li G., Wang P., Song C.P. (2020). BONZAI Proteins Control Global Osmotic Stress Responses in Plants. Curr. Biol..

[29] Yuan F., Yang H., Xue Y., Kong D., Ye R., Li C., Zhang J., Theprungsirikul L., Shrift T., Krichilsky B. (2014). OSCA1 mediates osmotic-stress-evoked Ca2+ increases vital for osmosensing in Arabidopsis. Nature.

[30] Stephan A.B., Kunz H.H., Yang E., Schroeder J.I. (2016). Rapid hyperosmotic-induced Ca2+ responses in *Arabidopsis thaliana* exhibit sensory potentiation and involvement of plastidial KEA transporters. Proc. Natl. Acad. Sci. USA.

[31] Wei J., Li D.X., Zhang J.R., Shan C., Rengel Z., Song Z.B., Chen Q. (2018). Phytomelatonin receptor PMTR1-mediated signaling regulates stomatal closure in *Arabidopsis thaliana*. J. Pineal. Res..

[32] Pelagio-Flores R., Munoz-Parra E., Ortiz-Castro R., Lopez-Bucio J. (2012). Melatonin regulates Arabidopsis root system architecture likely acting independently of auxin signaling. J. Pineal. Res..

[33] Gao F., Xie Y., Shen Y., Lei Z., Wang X., Xia H., Liang D., University S.A. (2018). Exogenous melatonin for NaCl stress with antioxidant enzymes and osmotic substances of *Aclinidia deliciosa* seedlings. J. Zhejiang A & F Univ..

[34] Kang K., Lee K., Park S., Kim Y.S., Back K. (2010). Enhanced production of melatonin by ectopic overexpression of human serotonin N-acetyltransferase plays a role in cold resistance in transgenic rice seedlings. J. Pineal. Res..

[35] Moustafa-Farag M., Almoneafy A., Mahmoud A., Elkelish A., Arnao M.B., Li L., Ai S. (2019). Melatonin and Its Protective Role against Biotic Stress Impacts on Plants. Biomolecules.

[36] Zhang J.R., Li D.X., Wei J., Ma W.N., Kong X.Y., Rengel Z., Chen Q. (2019). Melatonin alleviates aluminum-induced root growth inhibition by interfering with nitric oxide production in Arabidopsis. Environ. Exp. Bot..

[37] Zhang Y., Ji T.T., Li T.T., Tian Y.Y., Wang L.F., Liu W.C. (2020). Jasmonic acid promotes leaf senescence through MYC2-mediated repression of CATALASE2 expression in Arabidopsis. Plant Sci..

[38] Zhang Y., Tian Y.Y., Wang L.F., Li Y.H., Li T.T., Liu W.C. (2020). WDR5a functions in cadmium-inhibited root meristem growth by regulating nitric oxide accumulation in Arabidopsis. Planta.

[39] Li T.T., Liu W.C., Wang F.F., Ma Q.B., Lu Y.T., Yuan T.T. (2018). SORTING NEXIN 1 Functions in Plant Salt Stress Tolerance through Changes of NO Accumulation by Regulating NO Synthase-Like Activity. Front. Plant. Sci..

[40] Giannopolitis C.N., Ries S.K. (1977). Superoxide dismutases: I. Occurrence in higher plants. Plant Physiol..

[41] Aebi H. (1984). Catalase in vitro. Methods Enzymol.

[42] Liu W.C., Li Y.H., Yuan H.M., Zhang B.L., Zhai S., Lu Y.T. (2017). WD40-REPEAT 5a functions in drought stress tolerance by regulating nitric oxide accumulation in Arabidopsis. Plant Cell Environ..

