# Supplementary material for: CAND2/PMTR1 Is Required for Melatonin-Conferred Osmotic Stress Tolerance in Arabidopsis
Source: Int J Mol Sci. 2021 Apr 13;22(8):4014. doi: 10.3390/ijms22084014 (PMC8069227; doi:10.3390/ijms22084014)
Supplement: Supplementary file 1 [file ijms-22-04014-s001.pdf]

Supplemental Table S1

List of the primers used in this study.

Primer name	Sequence (5' to 3')
**Primers used for qRT-PCR**	
SNAT1-QF	GCCAAGGAGACCGTTAGTGA
SNAT1-QR	CCGCCTTCTGATGAATCTGAAT
COMT1-QF	ACTGGAGTGACGAACATTGC
COMT1-QR	GAGGCTTGAGTCTGGTGTCT
ASMT1-QF	TGATGGCGTGACTACGATGG
ASMT1-QR	CAACATTCTCAACACCGTCCAA
CAT1-QF	CTGCTCTGGAAATCGTGAGA
CAT1-QR	CGAATCGTTCTTGCCTGTCT
CAT2-QF	TCAAACCATGGATCCTTACAAGT
CAT2-QR	TGTTCCATACAGGAGCACCA
CAT3-QF	AAGCCTATTTGGGGGATCAT
CAT3-QR	TTGTACGCGCTTGAAGGAC
SOD1-QF	TGGAACTGCCACCTTCACAATCAC
SOD1-QR	CCAATGATGCCGCAAGCAACAC
ACTIN2/8-F	GGTAACATTGTGCTCAGTGGTGG
ACTIN2/8-R	AACGACCTTAATCTTCATGCTGC
